# Carbonation of Calcined Clay Dolomite for the Removal of Co(II): Performance and Mechanism

**DOI:** 10.3390/jox16010013

**Published:** 2026-01-13

**Authors:** Can Wang, Jingxian Xu, Tingting Gao, Xiaomei Hong, Fakang Pan, Fuwei Sun, Kai Huang, Dejian Wang, Tianhu Chen, Ping Zhang

**Affiliations:** 1 School of Environment and Energy Engineering, Anhui Jianzhu University, Hefei 230601, China; wangcan@ahjzu.edu.cn (C.W.);; 2 Key Laboratory of Environmental Pollution Control and Resource Reuse, Anhui Jianzhu University, Hefei 230601, China; 3 Key Laboratory of Nano-Minerals and Pollution Control of Anhui Higher Education Institutes, Hefei University of Technology, Hefei 230009, China; 4 College of Civil Engineering, Anhui Jianzhu University, Hefei 230601, China; 5 College of Civil Engineering, Bengbu University, Bengbu 233030, China

**Keywords:** cobalt pollution, remediation strategies, carbonation, mineral materials, clay dolomite

## Abstract

The rising levels of Co(II) in aquatic environments present considerable risks, thereby necessitating the development of effective remediation strategies. This study introduces an innovative pre-hydration method for synthesizing carbonated calcined clay dolomite (CCCD) to efficiently remove Co(II) from contaminated water. This pre-hydration treatment successfully reduced the complete carbonation temperature of the material from 500 °C to 400 °C, significantly enhancing energy efficiency. The Co(II) removal performance was systematically investigated by varying key parameters such as contact time, initial Co(II) concentration, pH, and solid/liquid ratio. Optimal removal was achieved at 318 K with pH of 4 and a solid/liquid ratio of 0.5 g·L^−1^. Continuous flow column experiments confirmed the excellent long-term stability of CCCD, maintaining a consistent Co(II) removal efficiency of 99.0% and a stable effluent pH of 8.5 over one month. Isotherm and kinetic models were used to empirically describe concentration-dependent and time-dependent uptake behavior. The equilibrium data were best described by the Langmuir model, while kinetics followed a pseudo-second-order model. An apparent maximum removal capacity of 621.1 mg g^−1^ was obtained from Langmuir fitting of equilibrium uptake data. Mechanistic insights from Visual MINTEQ calculations and solid phase characterizations (XRD, XPS, and TEM) indicate that Co(II) removal is dominated by mineral water interface precipitation. The gradual hydration of periclase (MgO) forms Mg(OH)_2_, which creates localized alkaline microenvironments at particle surfaces and drives Co(OH)_2_ formation. Carbonate availability further favors CoCO_3_ formation and retention on CCCD. Importantly, this localized precipitation pathway maintains a stable, mildly alkaline effluent pH (around 8.5), reducing downstream pH adjustment demand and improving operational compatibility. Overall, CCCD combines high Co(II) immobilization efficiency, strong long-term stability, and an energy-efficient preparation route, supporting its potential for scalable remediation of Co(II) contaminated water.

## 1. Introduction

Cobalt is a vital element in modern industry, with key roles in batteries, alloys, catalysts, and nuclear energy applications [1]. Its expanding production and use have increased the likelihood of environmental release. Discharge of cobalt-containing wastewater threatens aquatic ecosystems and human health because dissolved Co(II) can be assimilated by biota and transferred through the food web, inhibiting organism growth and reducing biodiversity [2]. Similar concerns extend to nuclear waste management, where cobalt, including radioactive cobalt, should be effectively immobilized within engineered barrier materials to limit long term release and exposure [3]. Sustained cobalt exposure has also been linked to adverse health outcomes, including respiratory impairment and neurological dysfunction [4]. Given these risks, effective strategies for cobalt pollution control and remediation are essential to mitigating its environmental and health impacts [5].

A range of technologies has been applied to Co(II) removal from water and soil, including membrane separation, chemical precipitation, ion exchange, and solid phase adsorption processes [6,7]. Among them, adsorption-based materials have been extensively explored because they can directly capture dissolved Co(II) without producing large volumes of chemical sludge under properly controlled conditions. Representative non-mineral adsorbents typically report Co(II) capacities on the order of 10 to 180 mg·g^−1^ [7]. For instance, oxidized activated carbon and carboxylated nanoporous graphene show removal values of 83.6 and 87.7 mg·g^−1^, respectively [8]. Membrane-type sorbents can further enhance capture efficiency, as exemplified by a GO BSA membrane with a reported Co(II) capacity of 180 mg·g^−1^ [9]. Despite these advances, many high-performance non-mineral adsorbents still face practical constraints for large-scale application, such as multi-step fabrication, reliance on costly precursors, and sensitivity of performance to water matrix complexity. These limitations motivate the development of low-cost mineral-derived media that can offer higher throughput Co(II) retention while maintaining manageable effluent chemistry.

Mineral-based materials are attractive in this context because they are abundant, inexpensive, and simple to operate at scale [10,11,12]. In addition to surface adsorption, mineral-based media can modulate aqueous chemistry during treatment by releasing alkalinity and providing carbonate buffering. Under suitable conditions, these geochemical functions may contribute to metal immobilization. They can generate elevated pH microenvironments at mineral-water interfaces, which may promote near-surface stabilization of metal-associated solids and reduce remobilization [13]. The magnitude of this effect is system-dependent and governed by mineral composition, dissolution kinetics, and local solution chemistry. This perspective motivates the development of abundant mineral precursors whose reactivity can be engineered to achieve high Co(II) retention while maintaining acceptable effluent pH. Clayey dolomite, a composite mineral mainly consisting of dolomite and attapulgite, represents a promising precursor in this regard. Nevertheless, raw clayey dolomite typically exhibits limited removal performance, restricting its direct use in heavy metal remediation [14]. Therefore, simple upgrading strategies are required to activate its reactivity and improve operational stability under realistic water treatment conditions.

Calcination is a straightforward activation route. During calcination, dolomite (CaMg(CO_3_)_2_) decomposes to generate lime (CaO)and periclase (MgO) (Equation (1)), which can markedly enhance Co(II) removal. Nevertheless, rapid CaO dissolution often elevates the effluent pH above 9.0, compromising long term applicability and increasing downstream neutralization demand. Carbonation of calcined clayey dolomite (CCCD) provides an alternative strategy by converting lime to calcite (CaCO_3_) (Equation (2)), thereby suppressing excessive alkalinity release and improving stability. This calcination and carbonation concept has proven effective for other divalent metals. Wang et al. [15] achieved 99.8% removal of 50 mg·L^−1^ Cd(II) within 1 h using calcined and carbonated dolomite, and Chen et al. [16] reported efficient Pb(II) fixation as hydrocerussite with a maximum capacity of 1150.06 mg·g^−1^, supported by gradual alkalinity and carbonate supply from CCCD. These results suggest that CCCD is a promising platform for Co(II)-contaminated waters, where both high removal and controlled effluent pH are required.

CaMg(CO_3_)_2_ → CaO + MgO + 2CO_2_ (1)

CaO + MgO + CO_2_ → CaCO_3_ + MgO (2)

Although carbonation is thermodynamically feasible at room temperature, kinetic barriers and product layer passivation often prevent full conversion under mild conditions. Therefore, conventional routes typically require carbonation temperatures near 500 °C to fully convert CaO, imposing a high thermal demand. Lowering the complete carbonation temperature can reduce the energy input and operating costs while limiting sintering and grain coarsening, thereby helping to preserve the porosity, reactive surface area, and long-term stability during use.

In this study, we developed a room temperature pre-hydration treatment that reduces the complete carbonation temperature of CCCD from 500 °C to 400 °C. We further show that high Co(II) removal can be achieved by regulating microscale interfacial alkalinity. This localized alkalinity promotes Co(II) fixation at mineral water interfaces. At the same time, alkalinity release to the bulk solution is limited. Therefore, the overall effluent remains only mildly alkaline (approximately pH 8.5). Co(II) removal performance was evaluated using batch tests and continuous flow column experiments. Mechanistic characterization was conducted to clarify the relationship among ion release, pH evolution, and interfacial Co(II) immobilization.

## 2. Materials and Methods

### 2.1. Materials

Clay dolomite (CD) was obtained from Zhenping, Henan Province, China. The sample was washed with deionized water to remove impurities and then dried at 105 °C for 12 h. The dried sample was crushed and sieved to particle sizes of 0.45–0.90 mm before being calcined at 860 °C for 1 h in a muffle furnace. After cooling, the calcined samples were stored in a sealed desiccator for further use and were labeled as CCD.

### 2.2. Carbonation Tests of the Calcined Clay Dolomite

The carbonation of calcined clay dolomite (CCCD) without pre-hydration was examined in a tubular furnace at 100 to 700 °C. In a typical run, 0.8 g of CCD was placed in a U-shaped quartz tube. The sample was exposed to a mixed gas of 10% CO_2_ and 90% Ar at 50 mL·min^−1^ for 2 h. For the pre-hydrated CCCD sample, CCD was conditioned in humid air (RH 60%) for 12 h at room temperature. It was then dried at 100 °C, followed by carbonation at temperatures ranging from 50 to 500 °C.

### 2.3. Removal Tests of Co(II) by CCCD

A standard solution of cobalt (Co(II), 1000 mg·L^−1^) was prepared by dissolving analytical-grade CoCl_2_·6H_2_O in deionized water. Batch experiments were conducted in 250 mL flasks. We added 50 mg of CCCD to 100 mL of Co(II) solution with initial concentrations of 10, 50, or 100 mg·L^−1^. The suspensions were stirred with a magnetic stirrer for 4 h.

For kinetic analysis, 3 mL aliquots were withdrawn at predetermined times (2, 5, 10, 20, 40, 60, 120, 180, 240, 300, and 360 min). The effect of solution pH was examined by adjusting the initial pH from 2 to 7 using 0.1 mol·L^−1^ HCl or 0.1 mol·L^−1^ NaOH. To investigate the effect of the solid-liquid ratio on Co(II) removal, different dosages of CCCD were added to 100 mL of Co(II) solution with a concentration of 50 mg·L^−1^. Equilibrium uptake isotherms and Co(II) removal were evaluated by varying the initial Co(II) concentration (10 to 100 mg L^−1^) under different temperatures (298 to 318 K). Co(II) concentration was determined using an atomic absorption spectrometer (AAS, Wanyee, WYS2000, China). Variations in the solution pH were recorded using a pH meter (Shanghai, Sanxin-SX-825, China). The Co(II) removal (R) and removal capacities (Q) were calculated using the following equations (Equations (3) and (4)).

(3) $$R=(c_{0}-c_{e})/c_{0}\times 100\%$$

(4) $$Q=\frac{(c_{0}-c_{e})V}{1000\;m}$$

where c_0_ and c*_e_* are the initial and equilibrium concentrations of Co(II), respectively. m is the sample weight.

A cylindrical glass reactor was used for the continuous flow experiment. A 5 cm layer of crushed glass was initially placed at the bottom of the reactor to serve as a support, followed by a 50 cm layer of CCCD. Another 5 cm layer of crushed glass was added on top of the CCCD, and the column was sealed with rubber stoppers. The reactor was positioned vertically, and silicone tubing was connected to both ends. Co(II) solutions were pumped from the bottom to the top of the reactor at a flow rate of 0.4 mL/min using a peristaltic pump.

### 2.4. Characterization

The chemical composition of the calcined clay dolomite was measured using an X-ray fluorescence spectrometer with Rh radiation (XRF, Shimadzu-1800, Kyoto, Japan). Prior to XRF analysis, the samples were pre-calcined at 800 °C to remove physically adsorbed water and minimize moisture-related interference, then cooled in a desiccator and ground into fine powders. The samples were characterized by X-ray diffraction (XRD) in a scan range of 3–70° with Cu Kα radiation (Dandong Haoyuan-DX-2007, Dandong, China). The tube voltage and current were set to 40 kV and 30 mA, respectively. The specific surface area (SSA), pore volume, and average pore diameter of the samples were measured using a surface area and pore size analyzer (Quanta Chrome NOVA 3000e, Florida, Boynton Beach, FL, USA). The surface morphology and elemental composition of the prepared sample before and after Co(II) removal were observed and analyzed using a transmission electron microscope (JEM-2100F, Akishima, Tokyo, Japan) with energy dispersive X-ray spectroscopy (EDS). The surface elemental composition of CCCD after Co(II) removal and the valence state of each element were analyzed using X-ray photoelectron spectroscopy (XPS, Thermo ESCALAB 250Xi, Waltham, MA, USA).

## 3. Results and Discussions

### 3.1. Components of Clayey Dolomite

**Figure 1:**
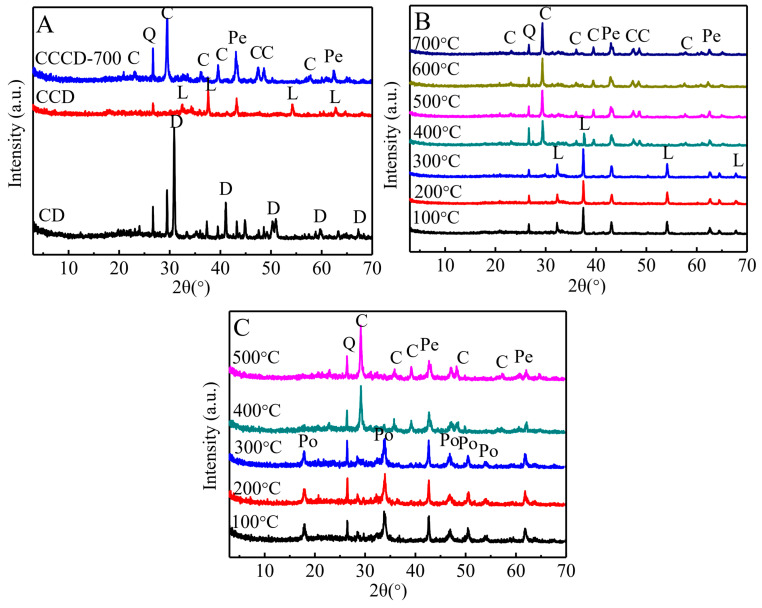
XRD patterns of the clay dolomite samples. (**A**) CD, CCD, CCCD-700; (**B**) CCD carbonation at different temperatures without hydration; (**C**) CCCD carbonation at different temperatures with hydration). (D-dolomite, L-lime, Q-quartz, Pe-periclase, C-calcite, Po-portlandite).

To establish the starting chemistry of the raw clayey dolomite, the bulk oxide compositions were first determined using XRF. The raw material contained CaO (31.14 wt.%), MgO (13.71 wt.%), SiO_2_ (10.7 wt.%), Al_2_O_3_ (2.54 wt.%), Fe_2_O_3_ (1.17 wt.%), others (0.83 wt.%), and a high ignition loss (39.91 wt.%). Because XRF provides bulk elemental information but does not resolve mineralogical host phases, XRD was subsequently employed to identify the dominant crystalline components and track phase transformations during calcination. As shown in Figure 1A, the XRD reflections of dolomite and quartz were clearly evident in the raw clay dolomite sample. However, the reflections corresponding to palygorskite are not discernible, presumably because the strong signals from dolomite overshadow those of palygorskite [15]. Considering the bulk chemistry and known associations of clayey dolomite, we infer that the raw material is dominated by dolomite, with subordinate palygorskite and quartz [17]. After calcination at 860 °C for 1 h, the dolomite decomposed completely, yielding lime (CaO) and periclase (MgO) in the CCD sample, as confirmed by the disappearance of the dolomite reflections and the emergence of CaO and MgO peaks.

### 3.2. Carbonated Product

The XRD patterns of the CCCD sample without pre-hydration carbonated under CO_2_ at different temperatures (100–700 °C) are shown in Figure 1B. Compared with the CCD sample (Figure 1A), there was no obvious change after carbonation when the temperature was below 300 °C. Above 400 °C, the reflection intensity of lime gradually decreased. Upon reaching 500 °C, the peaks of lime disappeared, while the reflection intensity of calcite, indicating complete carbonation at 500 °C under these conditions. In addition, the peaks of periclase remained almost unchanged, and reflections of magnesite were not observed, indicating that periclase was not involved in the carbonation reactions.

The CCCD sample underwent pre-processing hydration before carbonation to investigate its effect on the carbonation reaction. As shown in Figure 1C, periclase and portlandite are the predominant products at a carbonation temperature of 300 °C, with only a minor amount of calcite generated (Equations (5)–(7)). At 400 °C, the characteristic peaks of portlandite disappeared entirely and transformed into calcite (Equation (7)), signifying a complete carbonation reaction. Compared to the CCCD sample without hydration, the temperature required for complete carbonation decreased from 500 °C to 400 °C. This result indicates that converting lime to portlandite via pre-hydration lowers the temperature required for complete carbonation of the material.

CaO + H_2_O → Ca(OH)_2_ (5)

CO_2_ + H_2_O → H_2_CO_3_ (6)

Ca(OH)_2_ + H_2_CO_3_ → CaCO_3_ + 2H_2_O (7)

### 3.3. Batch Experiments for Co(II) Removal

#### 3.3.1. Cobalt Removal Performance

**Figure 2:**
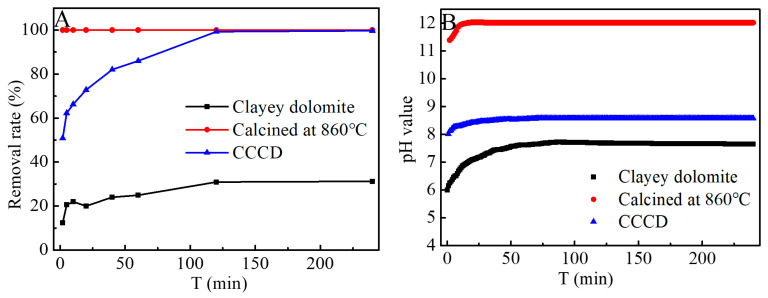
(**A**) The removal efficiency of Co(II) by CD, CCD, and CCCD, and (**B**) the real-time pH changes in the solution.

Comparisons were made regarding the efficacy of removing cobalt ions from raw clayey dolomite (CD), calcination clay dolomite at 860 °C (CCD), and hydration CCCD samples at 400 °C. As depicted in Figure 2A, when 50 mg of CD was introduced into a 50 mg·L^−1^ Co(II) solution, the reaction reached equilibrium after approximately 120 min, with a removal rate of approximately 31%. The pH of the solution varied following a similar trend, stabilizing at pH 7.6. A significant amount of blue-green precipitate was formed instantly after the addition of CCD to the cobalt solution. Analysis of the supernatant after membrane filtration revealed the absence of Co(II), indicating excellent cobalt removal by the CCD. However, the equilibrium pH was notably high at 12 (Figure 2B), which is attributable to the dissolution of CaO and MgO in the CCD, which releases abundant OH^−^, thereby promoting the precipitation of cobalt hydroxide species. For the CCCD sample, the Co(II) concentration gradually decreased with time and reached equilibrium, yielding a removal efficiency exceeding 99% at an equilibrium pH of 8.6 (Figure 2B).

#### 3.3.2. Effects of Initial pH and Solid-Liquid Ratio

**Figure 3:**
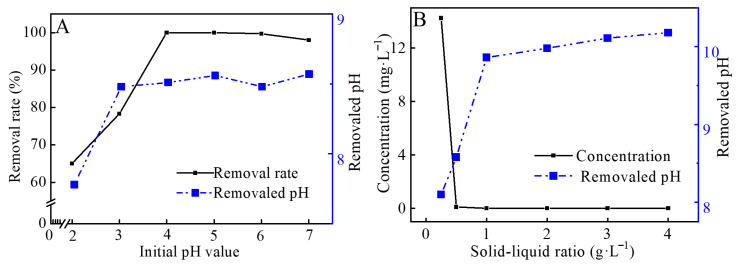
(**A**) Effects of initial pH and (**B**) solid-liquid ratios on Co(II) removal by CCCD.

The influence of different initial pH values on Co(II) removal by hydrated CCCD is shown in Figure 3A. The initial pH was adjusted from 2 to 7 to cover acidic to near-neutral conditions. When the initial pH increased from 2 to 4, the removal efficiency increased from 65.04% to over 99%. When the initial pH increased further to 6 and 7, the removal efficiency decreased slightly. The final pH values followed the same trend.

At pH 2 to 3, abundant H^+^ competes with Co(II) during surface interaction. The acidic environment also suppresses Co(II) hydrolysis and precipitation, which reduces overall immobilization. As pH increases, proton competition weakens and Co(II) hydrolysis becomes more favorable. At pH 4, the removal reaches its maximum. At pH 6 to 7, precipitation products can accumulate on particle surfaces and partially reduce accessibility, which explains the slight decrease in removal efficiency.

Figure 3B shows the effect of solid-liquid ratios on the removal of Co(II). The post-reaction concentration of Co(II) decreased to 14.22 mg·L^−1^ after 240 min when the solid-liquid ratio was 0.25 g·L^−1^. Increasing the ratio to 0.5 g·L^−1^ reduced the residual Co(II) to 0.10 mg·L^−1^, and further increasing the dosage to 1–4 g·L^−1^ resulted in no additional measurable decrease. Meanwhile, the final pH increased gradually with solid dosage and stabilized at approximately 9. Therefore, 0.5 g·L^−1^ was selected as the optimal solid to liquid ratio, achieving near complete removal with minimal material consumption.

#### 3.3.3. Kinetic

**Figure 4:**
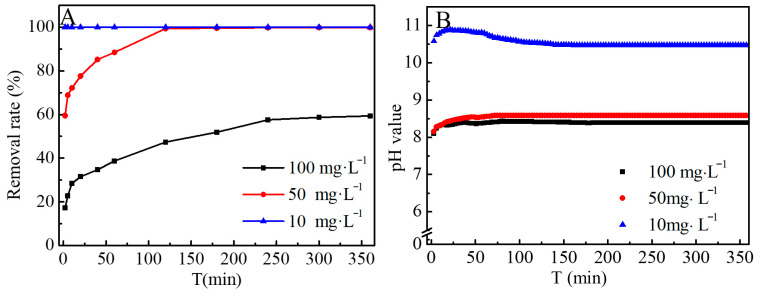
(**A**) The removal efficiency of CCCD for Co(II) with different initial concentrations, (**B**) the pH variation in the solutions.

For screening and kinetic evaluation, 10, 50, and 100 mg·L^−1^ were selected to represent low to moderate loadings. A 360 min test was used to confirm the approach to steady state. As shown in Figure 4A, equilibrium was reached quickly for 10 mg·L^−1^ Co(II) after adding hydrated CCCD. For 50 mg·L^−1^ Co(II), equilibrium was achieved at around 120 min and the removal rate exceeded 99%. For 100 mg·L^−1^ Co(II), equilibrium was reached at approximately 360 min and the removal rate decreased to about 60%. Overall, the time to reach equilibrium increases with the initial Co(II) concentration.

Figure 4B shows the pH evolution during the reaction. The pH increased rapidly at early times and then stabilized. The final pH values were approximately 10.5, 8.6, and 8.4 for 10, 50, and 100 mg·L^−1^ Co(II), respectively. This trend reflects alkalinity release from CCCD and alkalinity consumption by Co(II) hydrolysis and precipitation. At higher Co(II) loadings, alkalinity consumption is stronger and the pH stabilizes near the carbonate buffer range.

The removal process of CCCD on Co(II) was fitted using pseudo-first-order and pseudo-second-order kinetic models [18,19]. The pseudo-first-order model equation is expressed in Equation (8) [20]:

(8) $$\ln(q_{e}-q_{t})=\ln q_{e}-k_{1}t$$

The pseudo-second-order model equation is described in Equation (9) [21]:

(9) $$t/q_{t}=1/k_{2}\cdot {q_{e}}^{2}+t/q_{e}$$

where q_t_ and q_e_ are the uptake capacity (mg·g^−1^) at time t (min) and adsorbent at equilibrium capacity (mg·g^−1^), respectively. k_1_ is the rate constant in min^−1^ of pseudo-first-order model, and K_2_ is the rate constant in g·mg^−1^·min^−1^ of pseudo-second-order model.

As shown in Table 1, compared to the pseudo-first-order model, the pseudo-second-order model provides a better fit to the data. This indicates that the entire reaction process is controlled by surface-mediated Co(II) removal kinetics.

#### 3.3.4. Equilibrium Isotherm Models

The Langmuir model is widely used to describe saturation-type equilibrium uptake on an ideal homogeneous surface [22], and its expression is given in Equation (10):

(10) $$c_{e}/q_{e}=c_{e}/q_{m}+1/K_{L}q_{m}$$

where q_m_ is the maximum removal capacity (mg·g^−1^), K_L_ is the Langmuir equilibrium constant.

The Freundlich model is commonly applied to equilibrium uptake on heterogeneous surfaces [23], as described in Equation (11):

(11) $${\mathrm{logq}}_{e}={\mathrm{logK}}_{F}+(1/n){\mathrm{logc}}_{e}$$

where K_F_ and n are Freundlich constants.

**Figure 5:**
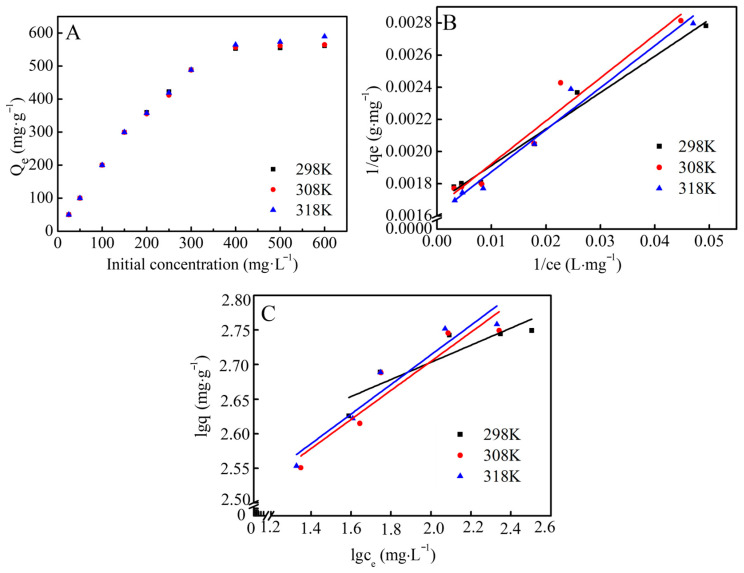
(**A**) Effect of initial concentration and temperature on the removal capacity of CCCD for Co(II); (**B**) fitted linear form of Langmuir isotherm plot; (**C**) fitted linear form of Freundlich isotherm plot.

Equilibrium isotherms were collected by varying the initial Co(II) concentration from 25 to 100 mg·L^−1^ at 298 to 318 K with an equilibration time of 24 h (Figure 5A). The equilibrium data were fitted using the Langmuir and Freundlich models (Figure 5B,C), and the corresponding parameters are listed in Table 2.

The mechanistic results presented in Section 3.4 suggest that Co(II) retention by CCCD is strongly influenced by precipitation-related interfacial processes and is not governed solely by classical adsorption onto fixed surface sites. Therefore, the Langmuir and Freundlich equations are employed here as empirical models to describe equilibrium uptake as a function of aqueous concentration. Although these models were originally developed for adsorption, in the present system they are used only to quantify concentration-dependent uptake behavior and to report an apparent maximum removal capacity for comparison.

As shown in Table 2, the Langmuir model yielded consistently higher R^2^ than the Freundlich model, indicating that the Langmuir equation provides a better empirical description of the equilibrium uptake behavior of Co(II) by CCCD under the present conditions. Notably, the maximum removal capacity calculated from the Langmuir model reached 621.1 mg·g^−1^ at 318 K.

The Langmuir fitting indicates a high Co(II) uptake capacity of CCCD (Q_max_ = 621.1 mg·g^−1^ at 318 K). As summarized in Table 3, this value is markedly higher than that of a representative carbon-based sorbent (oxidized activated carbon, 83.6 mg g^−1^) [8] and a membrane-type material (GO BSA membrane, 180 mg g^−1^) [9]. Because Co(II) retention by CCCD is mainly associated with precipitation-influenced interfacial processes rather than classical adsorption (Section 3.4), we also compared CCCD with a more mechanism-relevant alkaline oxide-based material, Ca-doped MgO. CCCD still exhibits a higher removal capacity than Ca-doped MgO (469.5 mg g^−1^) [24]. Collectively, these comparisons demonstrate that CCCD provides a favorable combination of high removal capacity and practical applicability for treating heavy metal-contaminated wastewater.

#### 3.3.5. Thermodynamic

Thermodynamic parameters were estimated from the temperature-dependent Langmuir constant (K_L_) obtained from equilibrium isotherm fitting at 298–318 K (Table 2). The apparent Gibbs free energy change (ΔG) at each temperature was calculated using Equation (12):

(12) $$\Delta G=-RTln\;K_{L}$$

where R is the universal gas constant and T is the absolute temperature (K). The apparent enthalpy (ΔH) and entropy (ΔS) were treated as overall parameters and obtained from the van’t Hoff regression of lnK_L_ versus 1/T using Equation (13). The corresponding regression coefficient is reported in Table 4.

(13) $$ln\;K_{L}=-\frac{\Delta H}{RT}+\frac{\Delta S}{R}$$

In this work, ΔG, ΔH, and ΔS are reported as apparent parameters derived from Langmuir fitting for comparative purposes. K_L_ decreases slightly with increasing temperature (0.0746 to 0.0615 L·mg^−1^), while ΔG increases from 6.431 to 7.373 kJ·mol^−1^ over 298–318 K. The negative ΔH indicates an exothermic overall retention trend, which is consistent with temperature-dependent interfacial stabilization (Section 3.4). In addition, it should be noted that Langmuir-based thermodynamic analysis has inherent limitations in precipitation-influenced systems and is used here only as a descriptive and comparative tool.

### 3.4. The Removal Mechanism of Cobalt by CCCD

**Figure 6:**
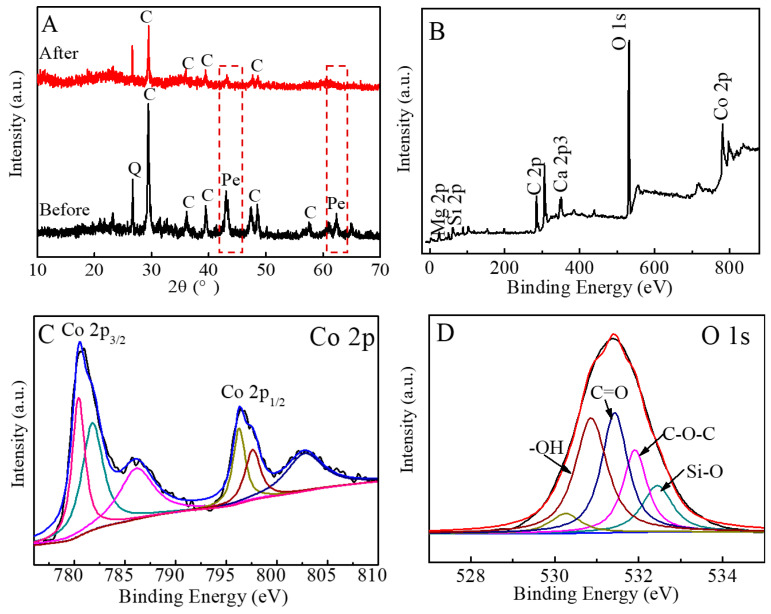
(**A**) XRD patterns of CCCD before and after Co(II) removal (Pe-periclase, C-calcite); (**B**) XPS full spectrum of CCCD after Co(II) removal; (**C**) Co 2p and O 1s (**D**) spectrum of CCCD after Co(II) removal.

As shown by the XRD results in Figure 6A, the characteristic diffraction peak of periclase (red dashed square) in the post-reaction CCCD sample nearly disappeared, and the intensity of the calcite peaks also decreased. This suggests that both periclase and calcite underwent dissolution and participated in the Co(II) removal process, which consequently contributed to the increase in solution pH after the reaction.

The post-reaction XPS spectrum of CCCD (Figure 6B) confirms the presence of Co, Ca, Mg, Si, C, and O. The Co 2p region shows major peaks at 780.4, 781.8, 796.3, and 797.6 eV, along with two minor shake-up features at 786.1 and 802.7 eV originating from the high-spin state of Co(II) (Figure 6C). The peaks at 781.8 and 797.6 eV correspond to Co 2p_3_/_2_ and Co 2p_1_/_2_ in Co(OH)_2_, whereas the peaks at 780.4 and 796.3 eV are attributed to Co^2+^ incorporated into carbonate (CoCO_3_) or silicate environments (such as palygorskite) [33,34]. These features indicate that Co^2+^ precipitated on the CCCD surface as Co(OH)_2_, and CoCO_3_ with part of the Co adsorbed onto silicate minerals. Additionally, the O 1s spectrum exhibited peaks at binding energies of 530.8 eV, 531.4 eV, 531.9 eV, and 532.4 eV, corresponding to -OH, C=O, C-O-O, and Si-O, respectively (Figure 6D) [35,36,37]. These oxygen environments are consistent with the coexistence of hydroxide, carbonate, and silicate phases involved in Co(II) retention on CCCD.

**Figure 7:**
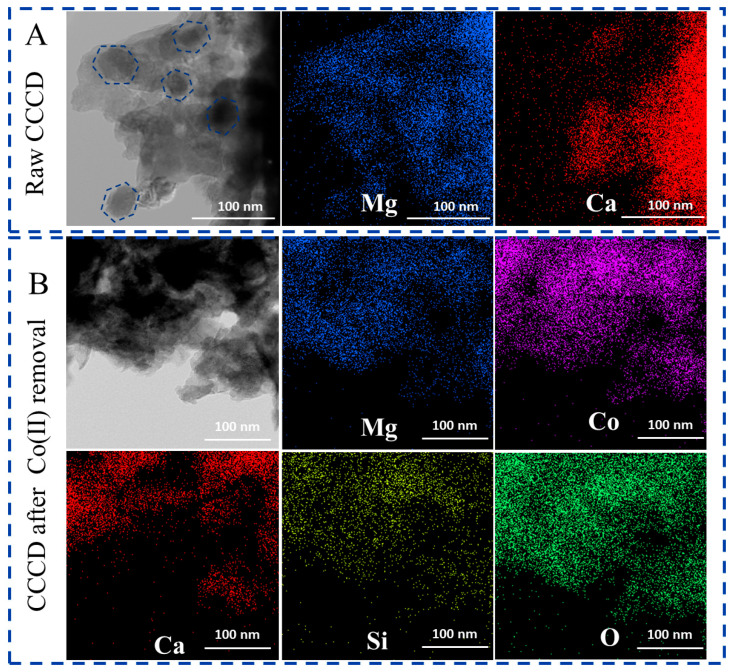
TEM images and corresponding EDS mapping of CCCD before (**A**) and after (**B**) Co(II) removal.

The TEM images and EDS mapping of CCCD before and after the reaction are shown in Figure 7. The carbonation of CCCD generated hexagonal nanosheet-like periclase domains, as evidenced by the Mg and Ca distributions in the EDS mapping (Figure 7A and Figures S1–S3). After Co(II) treatment, the Co signal was uniformly distributed across the CCCD surface(Figure 7B and Figures S4–S9). Together with the XPS results, this indicates that Co(II) is primarily retained on the CCCD surface in the form of Co(OH)_2_ and CoCO_3_ rather than precipitating in the bulk solution.

**Figure 8:**
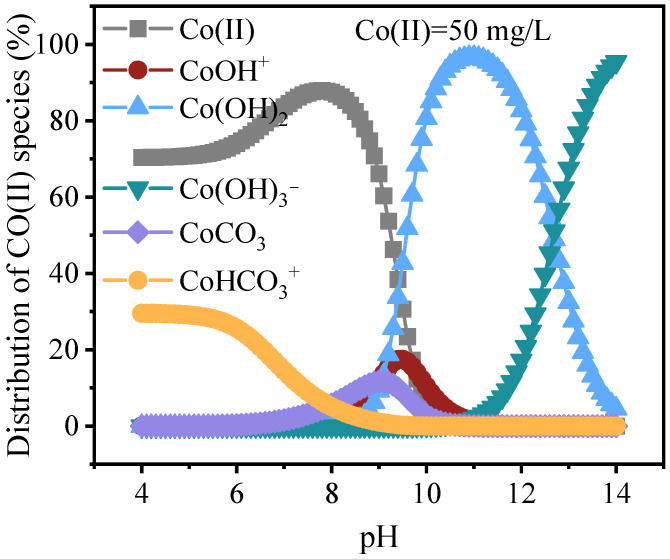
Simulation results of Co(II) species distribution via Visual MINTEQ software.

Because the batch experiments (Figure 3A) produced relatively high effluent pH values (approximately 8.5), it was necessary to evaluate the dominant Co(II) retention route. Three possibilities were considered: (i) surface physical adsorption, (ii) bulk precipitation in solution, and (iii) precipitation and retention on CCCD surfaces. To clarify this, the pH-dependent distribution of Co(II) species was calculated using the Visual MINTEQ model across a pH range of 4.0 to 14.0. As shown in Figure 8 at the representative effluent pH of 8.5, Co(II) occurs predominantly as the free ionic species (83.2%). Co(OH)_2_ and CoCO_3_ represent only 1.2% and 8.4%, respectively. These results indicate that extensive bulk precipitation in solution is unlikely under the experimental conditions. In addition, the removal cannot be attributed solely to physical adsorption. This is evidenced by the exceptionally high Co(II) removal capacity of CCCD (621.1 mg·g^−1^), which far exceeds that of activated carbon-based materials reported for conventional adsorption processes (typically 5 to 180 mg·g^−1^; Table 3).

Because neither bulk precipitation in the aqueous phase nor physical adsorption can adequately explain the observed removal efficiency, Co(II) uptake by CCCD is likely dominated by an interfacial precipitation process. Kawano and coworkers [13] showed that alkaline minerals such as calcite can generate strong pH gradients. The pH at the mineral water interface can reach values up to about 2 units higher than the bulk solution. Therefore, even though the measured effluent pH was approximately 8.5, the local pH at the mineral water interfaces of calcite and periclase within the CCCD could plausibly approach approximately 10.5. At such elevated interfacial pH, the Co(II) speciation calculations (Figure 8) indicate that Co(II) would predominantly occur as Co(OH)_2_ and CoCO_3_. Taken together, these results support the interpretation that Co(II) removal by CCCD is governed mainly by the microscale interfacial pH environment at mineral water interfaces rather than by precipitation in the bulk solution or simple adsorption.

**Figure 9:**
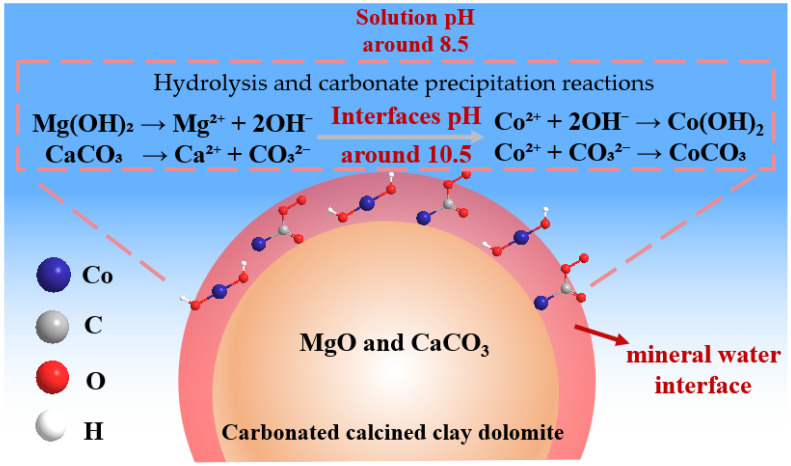
The proposed Co(II) removal of CCCD mechanism.

Based on these analysis results, the removal mechanism of Co(II) ions by CCCD can be inferred as follows (Figure 9). XRD and TEM analyses indicated that the calcination carbonation of the clay dolomite precursor yielded a composite material composed primarily of nanocrystalline periclase (MgO) and calcite (CaCO_3_). Upon contact with Co(II), both phases undergo partial dissolution at the mineral-water interface, releasing OH^−^ and CO_3_^2−^, according to the following reactions:

Mg(OH)_2_ → Mg^2+^ + 2OH^−^ (14)

CaCO_3_ → Ca^2+^ + CO_3_^2−^ (15)

These dissolution processes elevate the interfacial pH well above that of the bulk solution, creating a localized alkaline environment that drives subsequent Co(II) transformation. XPS results confirmed that Co(II) was predominantly retained as Co(OH)_2_ and CoCO_3_ on the CCCD surfaces, consistent with hydrolysis and carbonate precipitation reactions:

Co^2+^ + 2OH^−^ → Co(OH)_2_ (16)

Co^2+^ + CO_3_^2−^ → CoCO_3_ (17)

Collectively, these interfacial dissolution and precipitation pathways immobilize Co(II) directly on CCCD particles and account for the high removal efficiency observed experimentally. Co(II) precipitation is governed by localized alkalinity at mineral water interfaces rather than by elevated bulk solution pH. Therefore, the effluent remains only mildly alkaline (approximately 8.5). Such moderate pH conditions are beneficial for downstream treatment and facilitate environmentally compliant discharges.

### 3.5. Long-Term Continuous-Flow Performance of CCCD

**Figure 10:**
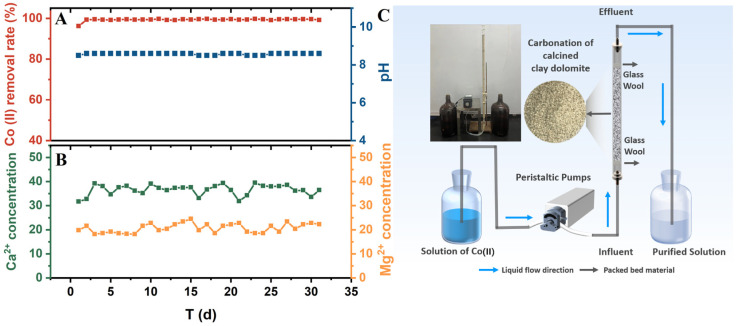
(**A**) Co(II) removal efficiency and effluent pH in the continuous flow reactor, (**B**) Ca^2+^ and Mg^2+^ concentrations in the reactor effluent, and (**C**) schematic diagram of the experimental setup.

To further evaluate the stability of CCCD and verify the proposed mechanism under dynamic conditions, a continuous flow experiment was conducted (Figure 10). The steady release of Ca^2+^ (approximately 35 mg·L^−1^) and Mg^2+^ (approximately 20 mg·L^−1^) during operation (Figure 10B) indicates sustained but controlled dissolution of calcite and periclase. This dissolution supplies OH^−^ and CO_3_^2−^ and helps maintain interfacial alkalinity. It also drives the formation of Co(OH)_2_ and CoCO_3_ at mineral water interfaces. Consistent with this controlled dissolution behavior, the effluent pH remained stable at approximately 8.5 over one month of operation (Figure 10A). The Co(II) removal efficiency remained at approximately 99.0%. These results support the conclusion that Co(II) transformation is governed by localized interfacial alkalinity rather than bulk solution pH elevation. The coherence between ion release, pH evolution, and removal performance demonstrates that CCCD maintains its reactivity under extended flow-through conditions. The moderate effluent pH also indicates good compatibility with downstream treatment steps.

## 4. Conclusions

This study developed a pre-hydration-assisted route to synthesize carbonated calcined clay dolomite (CCCD) and evaluated its application for Co(II) removal from contaminated water. The main conclusions are as follows:

(1) The pre-hydration treatment substantially enhanced carbonation efficiency. It lowered the temperature required for complete carbonation from 500 °C to 400 °C. This provides a more energy-efficient preparation strategy.
(2) CCCD exhibited excellent Co(II) removal performance under weakly acidic conditions (initial pH 4, 318 K). The apparent maximum removal capacity reached 621.1 mg·g^−1^. Continuous flow column tests verified strong long-term stability. The system maintained approximately 99.0% removal efficiency and a stable effluent pH of about 8.5 over one month.
(3) Visual MINTEQ calculations and solid phase characterizations (XRD, XPS, TEM) indicate that Co(II) immobilization is dominated by mineral water interface precipitation rather than extensive bulk precipitation at the measured effluent pH. The microscale alkaline environment at calcite and periclase surfaces promotes Co(OH)_2_ and CoCO_3_ formation and retention on CCCD particles, while maintaining a stable, mildly alkaline effluent.

Overall, CCCD provides an energy-efficient and scalable mineral-based option for Co(II) immobilization, with high capacity, month-scale stability under flow-through operation, and favorable effluent pH control, supporting its potential for practical treatment of heavy metal contaminated wastewater.

## Figures and Tables

**Table 1:** Pseudo-first-order and pseudo-second-order kinetic analysis for Co(II) removal by CCCD.

Concentration of Co(II) (mg·L^−1^)	Pseudo-First-Order			Pseudo-Second-Order		
	Q_e_ (mg·g^−1^)	K_1_	R^2^	Q_e_ (mg·g^−1^)	K_2_	R^2^
50	36.97	0.03	0.9829	72.3	0.0024	0.9995
100	73.65	0.01	0.9861	116.69	0.0004	0.9895

**Table 2:** Isotherm parameters calculated from Langmuir and Freundlich models for the equilibrium uptake of Co(II) by CCCD.

	Langmuir			Freundlich		
T/K	q_m_ (mg·g^−1^)	K_L_ (L·mg^−1^)	R^2^	K_F_ (mg·g^−1^)	1/n	R^2^
298	591.7	0.0746	0.9748	285.38	0.1239	0.757
308	602.4	0.0621	0.9419	192.32	0.2103	0.8607
318	621.1	0.0615	0.9679	192.95	0.2143	0.8958

**Table 3:** Comparative studies for the removal of Co(II) by various materials.

Adsorbent	Time (h)	pH	T (°C)	Co(II) (mg·L^−1^)	Dose (g·L^−1^)	Q_max_ (mg·g^−1^)	References
Oxidized activated carbon	2	4	/	50	0.03–0.1	83.6	[8]
Carboxylated nanoporous graphene	0.2	6	25	50	0.12	87.7	[25]
Fe_3_O_4_/bentonite	1.5	8	25	800	2	18.8	[26]
Apricot stone activated carbon	1.5	2–13.5	25–34	10–80	5–50	111.1	[27]
Amination graphene oxide nanocomposite	12	6	25	30	0.3	116.4	[28]
ZrO-Kaolinite	0.33–4	1–10	30–40	10–250	2–6	0.2	[29]
ZrO-montmorillonite	0.33–4	1–10	30–40	10–250	2–6	0.2	[29]
GO-BSA membrane	1.3	5–9	25–30	20–120	0.1	180	[9]
Doped glycerol	1.2	2–10	25	10–100	0.5–1	117.9	[30]
Ca-dopped MgO	0.3	1–8	/	5–200	0.4	469.5	[24]
Graphene oxide	1	5.5	25	2–25	1	21.3	[31]
Carbon gels	48	3–7	25	10–50	2	5.5	[32]
CCCD	6	4	25	10–100	0.5	621.1	This work

**Table 4:** Thermodynamic parameters for removal of Co(II) by CCCD.

T (K)	K_L_ (mg·g^−1^)	ΔG (kJ·mol^−1^)	ΔH (kJ·mol^−1^)	ΔS (J·mol^−1^)	R^2^
298	0.0746	6.431	−7.68	−47.57	0.8027
308	0.0621	7.117			
318	0.0615	7.373			

## Data Availability

The original contributions presented in this study are included in the article/Supplementary Material. Further inquiries can be directed to the corresponding authors.

## Supplementary Materials

The following supporting information can be downloaded at: https://www.mdpi.com/article/10.3390/jox16010013/s1, Figure S1: TEM of raw CCCD; Figure S2: Mg-EDS mapping of raw CCCD; Figure S3: Ca-mapping of raw CCCD; Figure S4: TEM of CCCD after Co(II) removal; Figure S5: Mg-EDS mapping of CCCD after Co(II) removal; Figure S6: Co-EDS mapping of CCCD after Co(II) removal; Figure S7: Ca-EDS mapping of CCCD after Co(II) removal; Figure S8: Si-EDS mapping of CCCD after Co(II) removal; Figure S9: O-EDS mapping of CCCD after Co(II) removal.
