# Supplementary material for: Carbonation of Calcined Clay Dolomite for the Removal of Co(II): Performance and Mechanism
Source: J Xenobiot. 2026 Jan 13;16(1):13. doi: 10.3390/jox16010013 (PMC12821731; doi:10.3390/jox16010013)
Supplement: Supplementary file 1 [file jox-16-00013-s001.zip › jox-4001134-supplementary.pdf]

Supplementary Materials: Carbonation of Calcined Clay Dolomite for the Removal of Co(Ⅱ): Performance and Mechanism

Can Wang, Jingxian Xu, Tingting Gao, Xiaomei Hong, Fakang Pan, Fuwei Sun, Kai Huang, Dejian Wang, Tianhu Chen and Ping Zhang

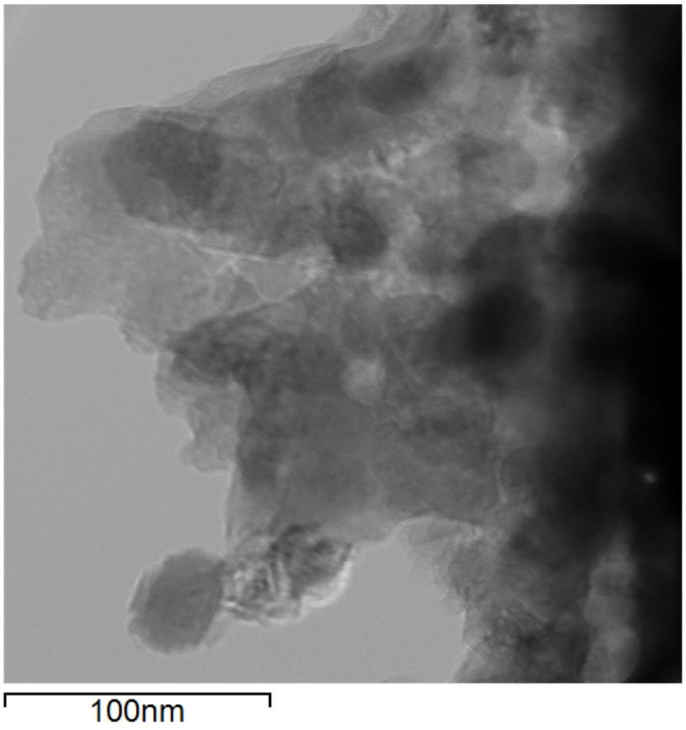

Figure S1. TEM of raw CCCD

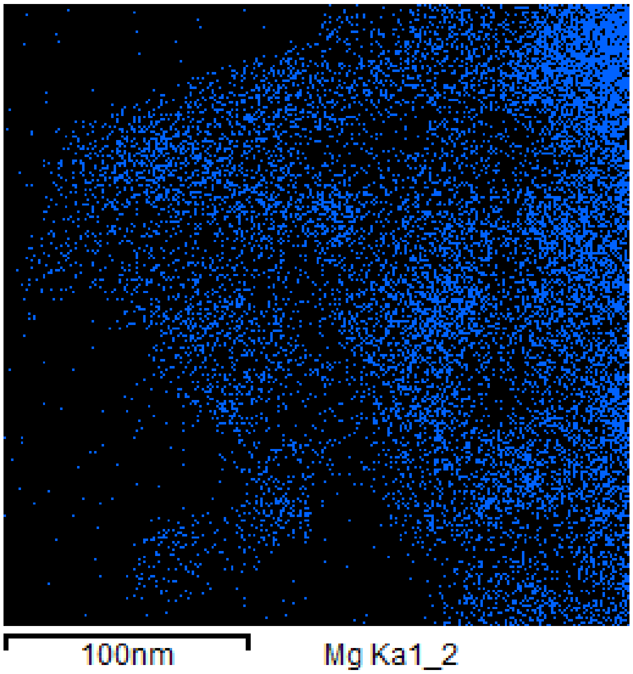

Figure S2. Mg-EDS mapping of raw CCCD

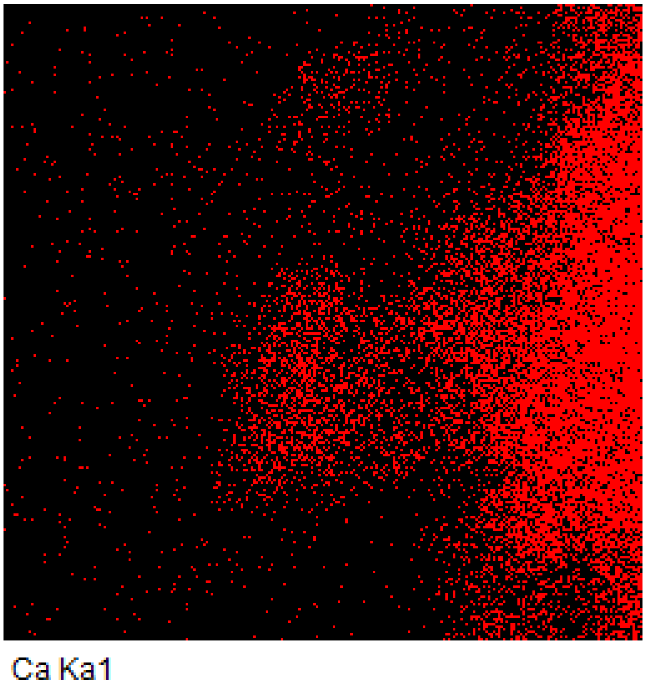

Figure S3. Ca- mapping of raw CCCD

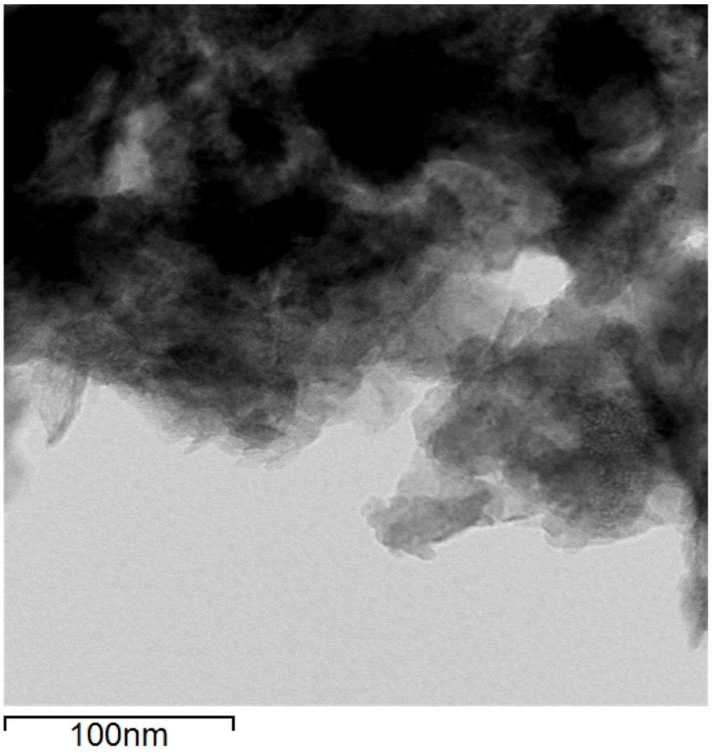

Figure S4. TEM of CCCD after Co(II) removal

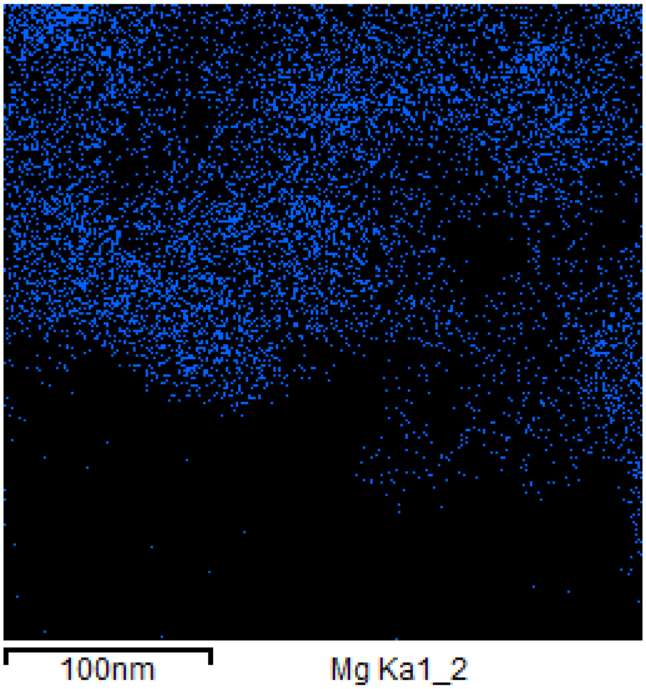

Figure S5. Mg-EDS mapping of CCCD after Co(II) removal

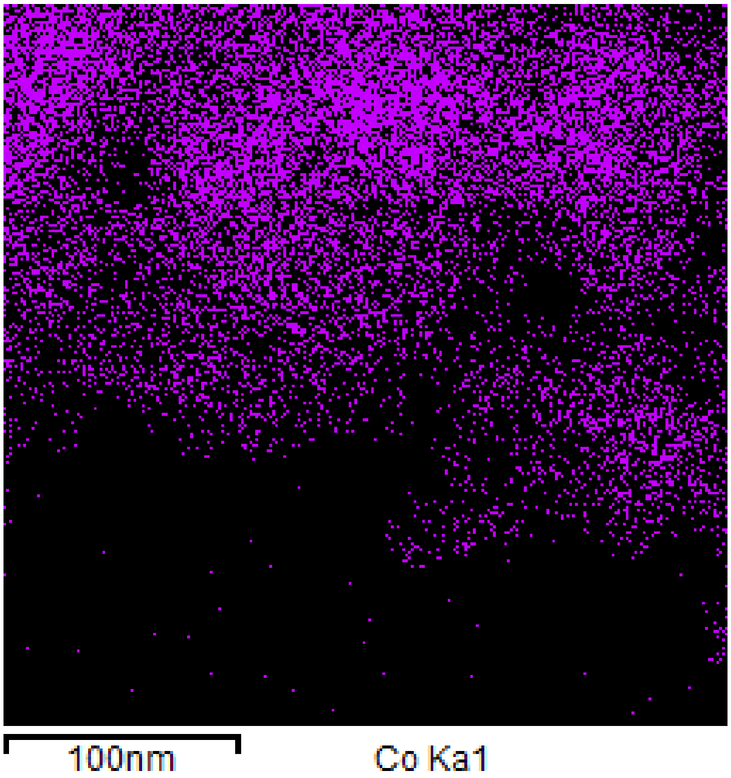

Figure S6. Co-EDS mapping of CCCD after Co(II) removal

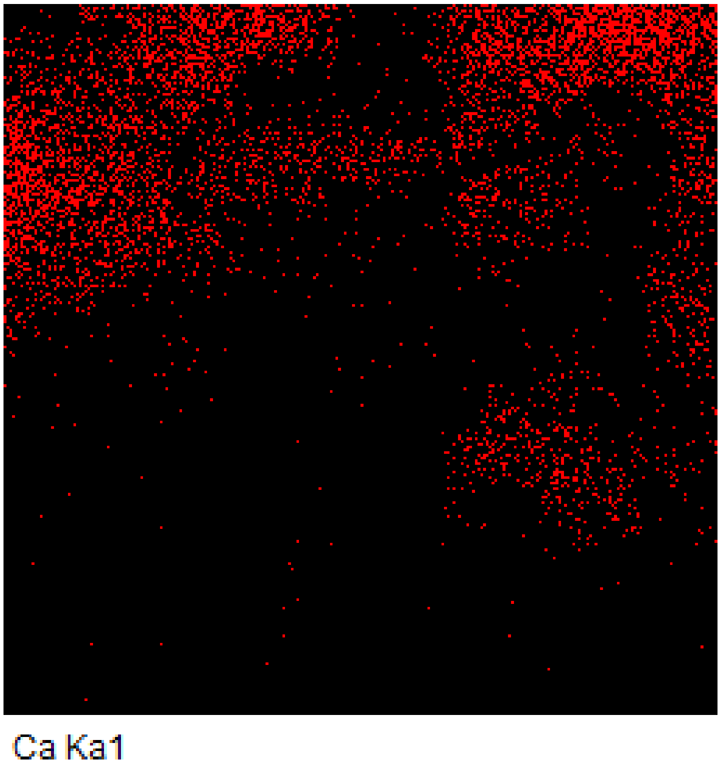

Figure S7. Ca-EDS mapping of CCCD after Co(II) removal

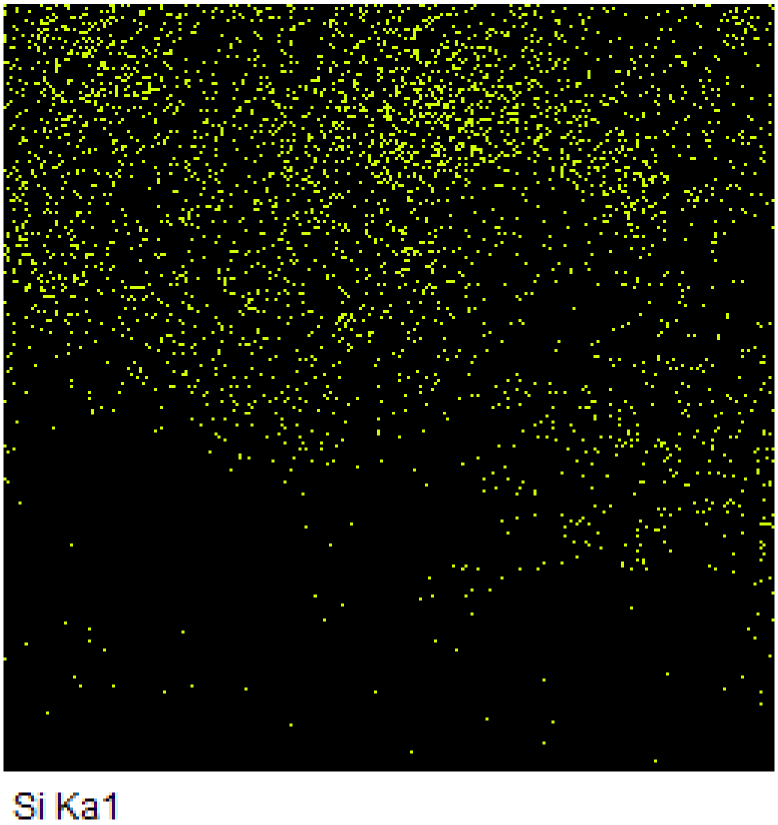

Figure S8. Si-EDS mapping of CCCD after Co(II) removal

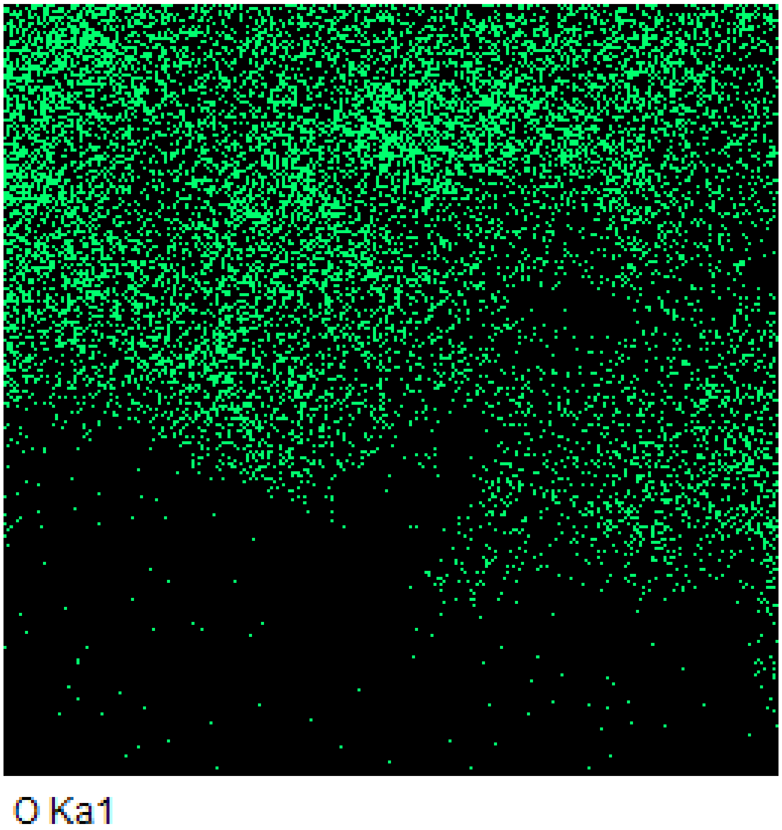

Figure S9. O-EDS mapping of CCCD after Co(II) removal
